# Evaluation of ecological green high-quality development based on network hierarchy model for the demonstration area in Yangtze River Delta in China

**DOI:** 10.3389/fpubh.2023.1159312

**Published:** 2023-03-13

**Authors:** Wen Wang, Lihong Chen, Xinfeng Yan

**Affiliations:** ^1^Shanghai International Fashion Science and Innovation Center, Donghua University, Shanghai, China; ^2^International Cultural Exchange School, Donghua University, Shanghai, China

**Keywords:** low carbon development, evaluation method, network hierarchy model, ecological green, evaluation index system

## Abstract

**Introduction:**

The construction of the Yangtze River Delta ecological green development demonstration area aims to take the lead in exploring an eco-friendly development model, demonstrating and leading the higher quality integrated development of the Yangtze River Delta in China.

**Methods:**

Through literature research, expert inquiries, and policy documents as the guidance, this study builds an ecological green high-quality development evaluation system for the demonstration area, including building an index system composed of 4 first-class indicators, 16 second-class indicators and 42 third-class indicators derived from economy, society and environment system, determining the index weight through the network analytic hierarchy process, and establishing the comprehensive evaluation index (CEI) and differential diagnosis index (DDI) of high-quality development, which is based on the relevant theory of statistical comprehensive index.

**Results:**

The establishment of this system provides a complete theoretical support and scientific guidance for the comprehensive evaluation of high-quality ecological green development and more balanced development of the demonstration area; and it can point out the development direction for the subsequent development of the Yangtze River Delta.

**Discussion:**

However, due to the availability of data, there is still room for further improvement in this paper. In the future research, the model can be used to evaluate the high-quality development level of the demonstration area through the relevant data of the demonstration area.

## 1. Introduction

The Yangtze River Delta region is a pioneer in China's economic development, social openness, innovation capacity and other aspects. It strives to build a unified market through regional integration, so as to form a joint force for development and promote high-quality development faster. The Yangtze River Delta Ecological Green Integrated Development Demonstration Zone (hereinafter referred to as the “Integrated Demonstration Zone”) includes three administrative districts and counties (hereinafter referred to as “two districts and one county”) under Shanghai, Jiangsu and Zhejiang. The construction of the integrated demonstration area aims to take the lead in exploring a development model, fully transform the advantages in the ecological field into the driving force of economic and social development, and finally feed back the ecological construction, so as to demonstrate and lead the comprehensive and high-quality development of the Yangtze River Delta. We took the innovation of regional integration system as a starting point to achieve common consultation, joint construction and sharing, as well as demonstrate and lead the higher quality integrated development of the Yangtze River Delta region. In the released Overall Plan for the Ecological and Green Integrated Development Demonstration Area in the Yangtze River Delta (hereinafter referred to as the “Overall Plan for the Demonstration Area”), the specific requirements for the construction of the integrated demonstration area in various fields have been deployed in detail, and the establishment of a high-quality statistical system of development level has been proposed. Therefore, according to the requirements of the plan, this paper constructs and applies a comprehensive evaluation system based on the high-quality development of the integrated development demonstration area, and quantitatively analyzes the results of high-quality development from the perspective of statistics. Therefore, this study intends to build a matching evaluation model for the high-quality development of the Yangtze River Delta region, and points out the direction for subsequent development based on this.

## 2. Connotation and connection of high-quality development, green development and regional integration

Ecological green integration demonstration area is the only demonstration area in China that covers both ecological green development goals and high-quality development goals (1). It is highly creative and innovative to take this demonstration area as the research object. Due to the short establishment time of the demonstration zone and the lag of relevant information release, the academic research on the integrated demonstration zone is still in its infancy. The existing research is mainly based on the official documents issued by the government, from the analysis of the development status of the integrated demonstration zone, the carding of the evaluation system and other aspects. In the existing research, some scholars closely followed the theme of “green development” of the demonstration area, evaluated the green development level of the demonstration area from three dimensions, including economic development, natural endowment and environmental governance, and diagnosed the advantages and disadvantages of green development of the demonstration area (2). Some scholars also understood the high-quality development of the demonstration area as a more adequate, balanced, comprehensive and sustainable development, and established an evaluation system to assess the construction level of the demonstration area around the overall strategic positioning and target requirements of the demonstration area (3). On the basis of building a high-quality development evaluation system, Jiaxing Municipal Bureau of Statistics carried out an empirical evaluation on the Jiaxian County (i.e., one of the “two districts and one county” in the demonstration area) under its jurisdiction, and put forward targeted countermeasures and suggestions (4). These research provide logical and methodological guidance for the in-depth study of the integrated demonstration zone, but there are still problems such as the evaluation system can not completely cover the development system, and the evaluation indicators can not be directly reflected in the evaluation results. Therefore, in order to build and apply the comprehensive evaluation system for high-quality development of the integrated demonstration area and promote the integrated demonstration area to build a high-quality development benchmark, we should first clarify the respective connotation and internal relationship of high-quality development, green development and regional integration, and then conduct comprehensive consideration to establish a comprehensive and scientific evaluation index system.

According to the relevant reports of the Chinese government and the statements made by national leaders, high-quality development is characterized by low input of production factors, high efficiency of resource allocation, low cost of resources and environment, and good economic and social benefits. Its purpose is to meet the growing needs of the Chinese people for a better life. In addition to the policy connotation, scholars also interpreted the definition of high-quality development in their respective research fields. In the previous studies, scholars mainly analyze the connotation of high-quality development from two perspectives. One is to analyze the comprehensiveness of high-quality development from the perspective of five major development concepts (innovation, coordination, green, openness and sharing). The other is to analyze the weaknesses in specific areas in the development process from the perspective of China's basic social contradictions at this stage, and high-quality development aims to complement these weaknesses (5). In addition, some scholars believe that the essence of the problem of high-quality development is to do with the problem of system construction, and goes on to emphasize that high-quality development is a comprehensive exposition supported by high-quality governance structure and oriented by high-quality society and high-quality economic coordination (6). In short, high-quality development is an efficient resource allocation and development mechanism committed to using fewer production factors to input and output higher economic and social benefits, while meeting the requirements of low resource and environmental costs. From this point of view, the connotation of high-quality development of the ecological integration demonstration area can be understood as an open construction mode that takes the green development of the ecological environment as the premise, the coordinated allocation of social elements as the basic, the active introduction of innovative resources as the driving force, and the joint construction and sharing of administrative regions as the starting point.

According to early data, the total national carbon footprint in 2017 was 299 × 107 tons, and the carbon emissions caused by production and consumption in the Yangtze River Economic Belt accounted for 85 to 95 percent of the total carbon emissions from consumption (7). The essence of green development is to diversify development goals, that is, to ensure the sustainability of natural resources while pursuing economic growth. It emphasizes the integrated and coordinated development of economic system, social system and natural system, 2014. Green development covers many fields such as current resource and energy conservation and efficient utilization, environmental pollution control, ecological restoration, circular economy, clean production, land and space planning, and is the basic way to break the constraints of resources and environment (8).

In early studies, there were practices similar to regional integration construction. For example, the Commonwealth of British West Indies Colonies was established in 1956, and its integrated administrative management system was considered to be able to effectively promote economic development (9). In the long run, regional integration is not only the general trend of global development, but also the endogenous needs of social and economic development (10). From the perspective of participants, regional integration includes government, enterprises, social organizations and other subjects. These subjects jointly participate in the integration process, in which the government plays a leading role. Through policy tools, barriers to communication and exchange within the region are eliminated, and a unified regional market is created to improve the overall welfare level of the region. It includes four stages: Trade integration, factor integration, policy integration and complete integration (11). From the perspective of cooperation content, regional integration is embodied as “functional integration” and “institutional integration”. Functional integration refers to the inter regional entities breaking communication barriers, complementing and relying on each other through trade exchanges, transportation and infrastructure; Institutional integration refers to the integration of rules, agreements, treaties, mechanisms, and policies to regulate and guide each other's behavior, so as to narrow the institutional gap (12). In short, regional integration is a complex system containing many factors, which needs to be considered from the perspective of economic and social development (10). Integration is closely related to regional social and economic development. For example, some scholars have proved through empirical research that regional integration can improve the overall quality of economic development of the Yangtze River Delta urban agglomeration (13).

In general, both high-quality development and green development are systematic expositions of social and economic development from macro, medium and micro perspectives. With the tightening of resource and environmental constraints in the Yangtze River Delta, taking green ecological integration as the guide, promoting higher quality economic integration has become an important way to solve social problems (14). The high-quality development of the integrated demonstration area should seize the advantages of ecological green and integration to achieve green economy, high-quality life and sustainable development.

## 3. Construction of evaluation system and selection of indices

Comprehensive evaluation is to establish an indicator system for specific research objects, make an overall judgment on the content to be evaluated, and achieve quantitative results. High quality development is a systematic and dynamic comprehensive concept, covering all kinds of subjects and elements in many fields such as ecology, economy and society, and there are complex internal relationships. This paper will combine the construction steps of comprehensive evaluation and carry out research according to the following steps: build a comprehensive high-quality development evaluation system and select representative indicators. Determine the weight of each indicator; Build a high-quality development model for the integrated demonstration area.

According to the overall plan of the demonstration area, the strategic objectives of the integrated demonstration zone are the transformation of ecological advantages, green innovative development, integrated system innovation, and harmony and livability between man and nature. In combination with the policy documents, the sorting out of concepts such as high-quality development in the previous text, and the development laws of mature urban agglomeration integration, this study aims at scientific guidance and reasonable evaluation of the construction of the demonstration area, positioning the objectives of high-quality development of the integrated demonstration area as: high-value ecological transformation, sustainable economic development, high-efficiency policy coordination and high-quality living environment. The target orientation also constitutes the main dimension of this study to evaluate the high-quality development of the integrated demonstration area.

High-value ecological transformation is the environmental guarantee for the high-quality development of the integrated demonstration area. The integrated demonstration area covers a total area of 2,413 square kilometers, including many water network lakes focusing on the “one river, three lakes” of the Taipu River, Dianshan Lake, Yuandang lake and FENHU lake. The water surface rate is 18.6%, and there are 396 lakes of all sizes. The river–lake relationship in the basin and the people around it constitute a community of Destiny (15). For the water ecosystem, cross-border collaborative governance has become a relatively mature governance paradigm (16). Therefore, it is necessary to give full account of the ecological and environmental advantages of the integrated demonstration area, explore and practice the development mode of ecological priority, green development and rural revitalization, and create a new benchmark for the transformation of ecological advantages. Environmental optimization can not only reduce carbon emissions, but also improve land use and public health, thereby contributing to the conservation of biodiversity and thus supporting the efficient operation of the carbon cycle. This dimension specifically includes ecological environment governance, ecological space construction and ecological culture development.

Sustainable economic development is both the capital guarantee and the key goal of high-quality development in the integrated demonstration zone. Innovation support is an important chain connecting ecological environment protection and high-quality economy (17). Green innovation is a community of circular promotion jointly formed by green development and innovation. Green innovation can not only promote regional economic growth, but also improve the overall social welfare level (18). The high-quality development of the integrated demonstration zone should rely on the existing natural, cultural, industrial and other high-quality resources in the demonstration zone, excavate and gather more innovative elements, cultivate the industrial innovation ecosystem, and form a regional green innovation development highland. The specific content of this dimension includes two aspects: the green economy business form and the innovative development level.

The coordinated policy system is the institutional guarantee for the high-quality development of the integrated demonstration zone. The realization of high-quality economic development needs the support of system (6). A systematic system can not only provide guidance for high-quality development participants, but also constrain their behavior. Integrated institutional innovation is an inevitable requirement for the high-quality development of the demonstration area. It focuses on institutional arrangements in planning management, ecological protection, land management, factor flow, fiscal and tax sharing, public services and other fields, forms policy synergy and coordination, and realizes integrated institutional innovation of common consultation, joint construction, common management, sharing and win-win. Policy coordination includes unified planning and management system, ecological environment protection system, land management mechanism, factor flow system, fiscal and tax sharing system, public service policy, credit management system among others.

High efficiency policy coordination is the institutional guarantee for the high-quality development of the integrated demonstration area. From the perspective of the overall development trend of interconnection among economy, society and system, the realization of high-quality economic development needs the support of system (6). A prominent feature of the high-quality development of the integrated demonstration area is the harmony and livability between man and nature. The two districts and one county in the integration demonstration area have always had close economic exchanges and cultural affinity, and the residents have a strong centripetal force and sense of unity. Existing studies have shown that the higher the degree of equalization of public resources and services such as regional medical care and education, the greater the social efficiency (17). Therefore, improving the infrastructure system and public service network, carrying forward the poetic Jiangnan water culture and promoting the integration of urban and rural development is one of the key ways for the high-quality development of the integrated demonstration area. The content of this dimension includes building the cultural brand of Jiangnan Water Town, beautiful and livable rural environment, connectivity infrastructure system, high-level public service guarantee and many others.

Focusing on the evaluation objective of high-quality development of the integrated demonstration area, according to the specific requirements of the Overall Plan of the Yangtze River Delta Ecological and Green Integrated Development Demonstration Area, as shown in Table 1, the first column includes four first level evaluation indicators of economy, society, environment and system, the second column includes 16 second level indicators, and the third column includes 42 third level indicators. The selection of indicators, measurement basis, measurement methods and other specific contents follow the principle of science, comprehensiveness and operability.

**Table 1:** High-quality development evaluation indicators of Yangtze River Delta ecological green integrated development demonstration area.

**Level-I**	**Level-II**	**Level-III**
High-value ecological transformation (environment) Env	Ecological environment management	Water Quality (Env_11_)
		Air quality (Env_12_)
	Construction of ecological space	Percentage of greenery coverage (Env_21_)
		Percentage of forest coverage (Env_22_)
	Ecological culture development	Construction process of ecological corridor (Env_31_)
		Development and utilization of ecotourism resources (Env_32_)
Sustainable economic development (economy) Eco	Green economy	The development of sports industry (Eco_11_)
		The development of new agricultural formats (Eco_12_)
		The development of green finance industry (Eco_13_)
	Innovation-driven development	Investment attraction of innovative enterprises (Eco_21_)
		Industry-university Research Cooperation (Eco_22_)
		Innovation space and platform construction (Eco_23_)
		Digital platform construction (Eco_24_)
Efficient policy coordination (system) Sys	Planning management system	Unity of land spatial planning (Sys_11_)
		Unity of planning management platform (Sys_12_)
	Ecological environment protection system	Unity of ecological protection laws and regulations (Sys_21_)
		Unity of ecological environmental standards (Sys_22_)
		Unity of ecological environment monitoring (Sys_23_)
		Unity of ecological environment law enforcement (Sys_24_)
	Land management system	Unity of construction land use planning (Sys_31_)
		Unity of the inventory land activation mechanism (Sys_32_)
		Unity of management of acquisition, storage and transfer of construction land (Sys_33_)
	Factor flow system	Unity of enterprise registration standards (Sys_41_)
		Unity of enterprise service supervision system (Sys_42_)
		Unity of mutual recognition and sharing of talent qualifications (Sys_43_)
		Unity of cross regional transaction of element resources (Sys_44_)
	Financial and tax management system	Unity of tax collection and management (Sys_51_)
		Unity of Cross Regional Sharing of Finance and taxation (Sys_52_)
	Public service policy system	Unity of public service standards and systems (Sys_61_)
		Unity of public service sharing (Sys_61_)
	Public credit management system	Unity of public credit evaluation system (Sys_71_)
		Unity of regional credit reward and punishment system (Sys_72_)
High-quality living environment (Society) Soc	Jiangnan Water Town cultural brand	Integrated development of humanity and nature (Soc_11_)
		Traditional culture development project (Soc_12_)
		Eco-cultural tourism development Soc_13_
	Beautiful and livable rural environment	Construction of beautiful countryside and boutique Village (Soc_21_)
		Rural ecological environment governance (Soc_22_)
	Interconnection infrastructure system	Construction of transportation network system (Soc_31_)
		New infrastructure construction (Soc_32_)
		Intelligent construction and Application (Soc_33_)
	High-level public service guarantee system	Supply of public service resources (Soc_41_)
		Facilitation level of social security services (Soc_42_)

## 4. Construction of the evaluation model

### 4.1. Determination of index weight

The evaluation of the high-quality development of the integrated demonstration area is a systematic and complex project. There is a relationship of mutual correlation and interaction among its evaluation indicators. For example, the planning and management system will affect the ecological and cultural development and the livable rural environment. The green economy format is also closely related to the relevant policies and systems. Other indicators also have similar correlation and impact. The analytic network process (ANP) is a further development of analytic hierarchy process (AHP). When there are many elements to be decided, and the relationship between the elements is complex, and network analytic hierarchy process can provide more optimized solutions.

This study studies whether the index layer is independent and interacts with each other, then it constructs a typical hierarchical structure, builds a judgment matrix by using expert scoring method, and then solves the super matrix. By using the super matrix, it comprehensively analyzes all the interacting and influencing factors to obtain their mixed weights, and constructs an evaluation index system.

(1) Analysis of correlation and influence of index factors

After consulting experts in relevant fields and analyzing relevant data, this study combs the influence relationship among the entire evaluation indicators, and constructs the correlation matrix table shown in Table 2, “1” in the table indicates that there is an association between the indicators in the left column and the corresponding indicators above the table.

**Table 2:** Correlation matrix between indices.

**Indicator type**		**High-value ecological transformation (environment) Env**			**Sustainable economic development (economy) Eco**		**Efficient policy coordination (system) Sys**							**High-quality living environment (Society) Soc**			
		**Ecological environment management**	**Construction of ecological space**	**Ecological culture development**	**Green economy**	**Innovation-driven development**	**Planning management system**	**Ecological environment protection system**	**Land management system**	**Factor flow system**	**Financial and tax management system**	**Public service policy system**	**Public credit management system**	**Jiangnan Water Town cultural brand**	**beautiful and livable rural environment**	**Interconnection infrastructure system**	**High- level public service guarantee system**
High- value ecological transformation (environment) Env	Ecological environment management							1						1	1		
	Construction of ecological space						1								1		
	Ecological culture development				1								1	1			
Sustainable economic development (economy) Eco	Green economy			1										1	1		
	Innovation-driven development											1					
Efficient policy coordination (system) Sys	Planning management system		1													1	
	Ecological environment protection system	1															
	Land management system															1	
	Factor flow system										1						1
	Financial and tax management system									1							
	Public service policy system					1											
	Public credit management system			1													
High-quality living environment (Society) Soc	Jiangnan Water Town cultural brand	1		1	1												
	Beautiful and livable rural environment	1	1		1												
	Interconnection infrastructure system						1		1								
	High- level public service guarantee system									1							

**Figure 1:**
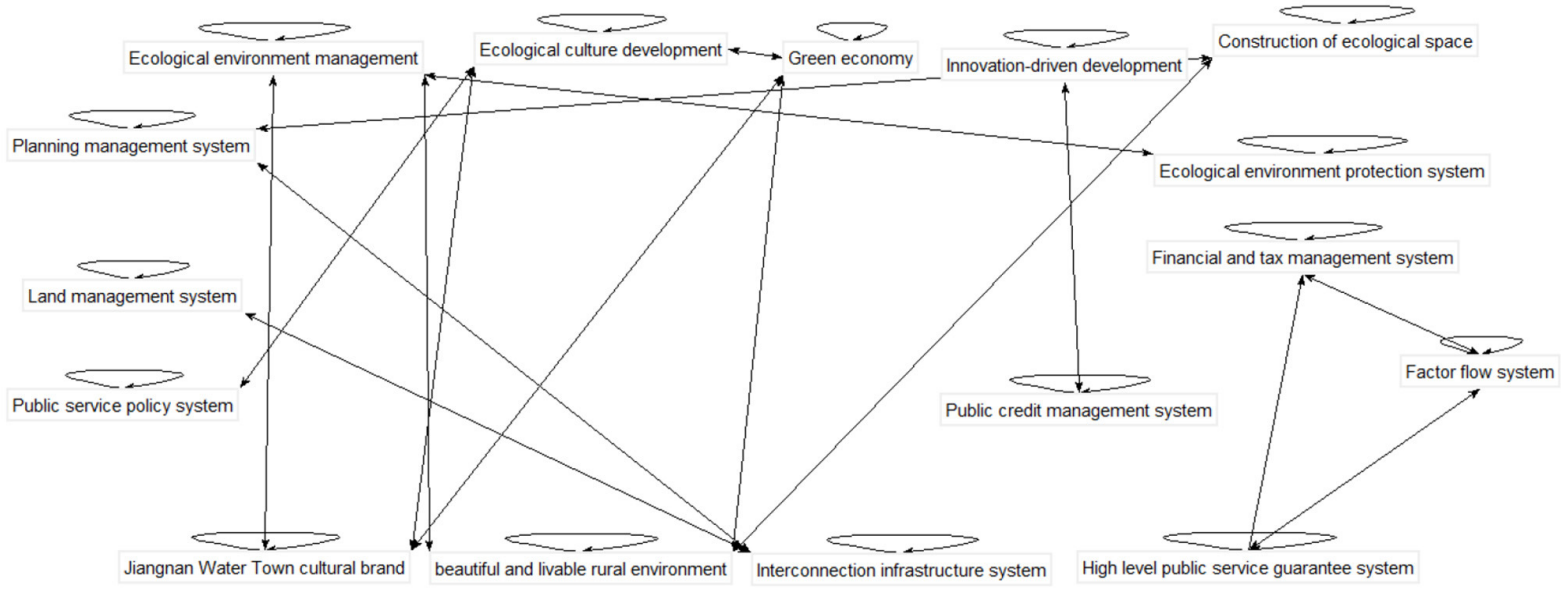
Index network diagram.

Based on the above matrix, the indicator network relationship built by super decisions software is shown in Figure 1.

(2) Construction of evaluation model based on ANP

**Figure 2:**
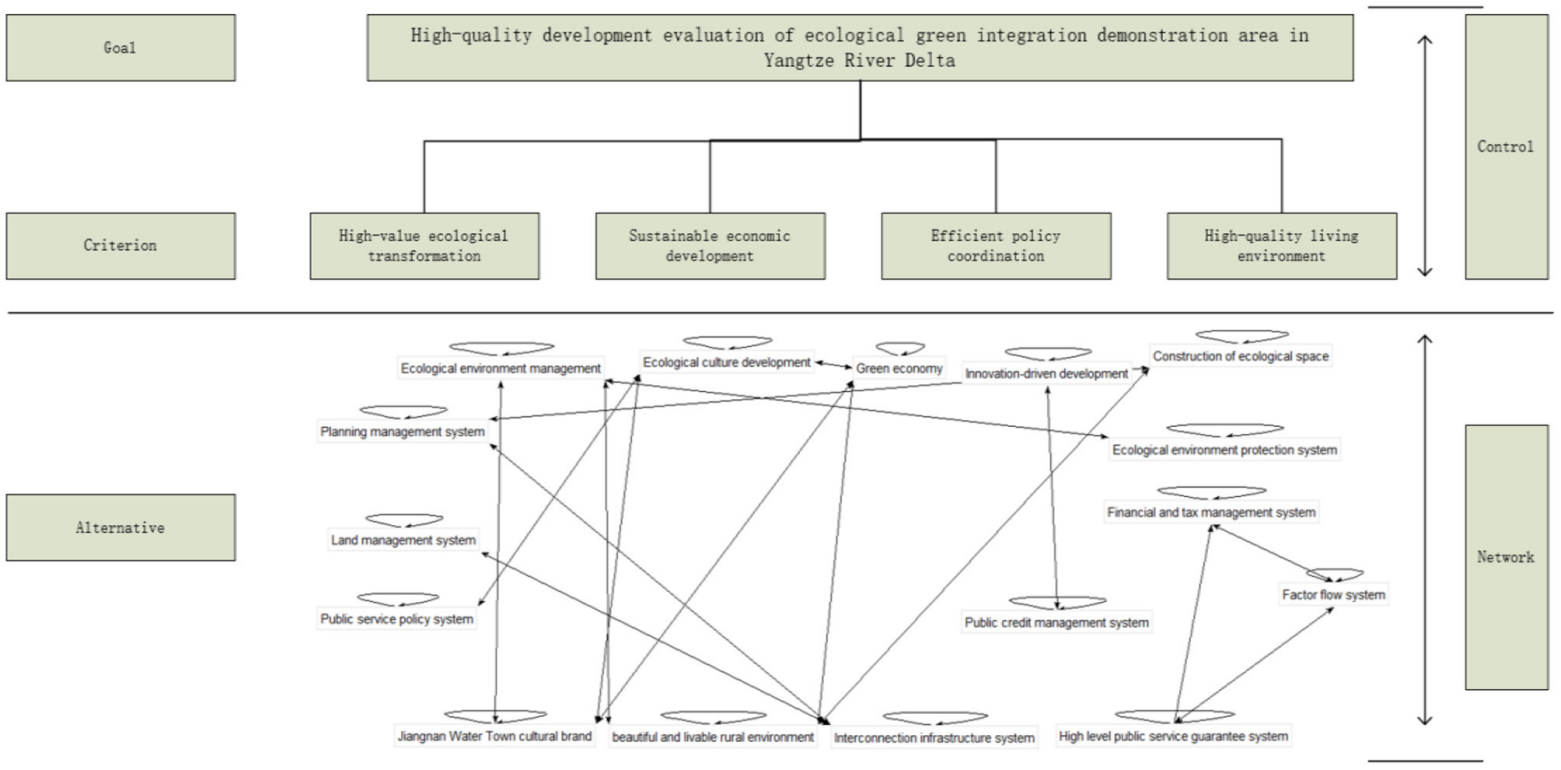
High-quality development evaluation model of Yangtze River Delta eco green integrated development demonstration area.

By analyzing the indicator system and the relationship between indicators, this paper builds a high-quality development evaluation model of the integrated demonstration area based on the network AHP method (Figure 2).

The elements of the index system of the evaluation model are divided into two parts: the control layer and the index layer. The control layer includes the target layer and the criterion layer. The indicators of the criterion layer and the criterion layer are relatively few, and the indicators are independent of each other. The traditional analytic hierarchy process can be used to obtain the indicator weight. The network layer includes 16 secondary indicators and their respective subordinate tertiary indicators, which are all affected by the control layer and interact with each other internally. Through the Super Decisions software, according to the principle of network level analysis, this study invited relevant experts and scholars to score the indicators based on the four criteria indicators (high value ecological transformation, sustainable economic development, efficient policy coordination, high-quality living environment) of the control layer. Since there are many index coefficients and the accuracy of subjective evaluation by experts is limited, this study invited experts from relevant industries to compare and score the element level indicators in pairs; The judgment matrix is constructed, and the consistency of the judgment matrix is checked. The consistency coefficient of the judgment matrix of each element and subset is < 0.1, and the consistency of the judgment matrix is passed.

After the consistency test is passed, the weighted super matrix is generated by constructing the unweighted super matrix and performing normalization analysis. Finally, the weighted super matrix is stabilized, and the weight results of each evaluation index can be obtained (Table 3).

**Table 3:** Weight of high-quality development evaluation indicators of integrated development demonstration area.

**Level-II**	**Weight**	**Level-III**	**Global weight**	**Local weight**
Ecological environment management	0.2252	Water Quality	0.1585	0.7040
		Air quality	0.0666	0.2960
Construction of ecological space	0.0271	Percentage of greenery coverage	0.0195	0.7189
		Percentage of forest coverage	0.0076	0.2811
Ecological culture development	0.0341	Construction process of ecological corridor	0.0231	0.6778
		Development and utilization of ecotourism resources	0.0110	0.3222
Green economy	0.0680	The development of sports industry	0.0157	0.2314
		The development of new agricultural formats	0.0315	0.4633
		The development of green finance industry	0.0208	0.3053
Innovation-driven development	0.1071	Investment attraction of innovative enterprises	0.0501	0.4673
		Industry-university Research Cooperation	0.0297	0.2772
		Innovation space and platform construction	0.0172	0.1601
		Digital platform construction	0.0102	0.0954
Planning management system	0.0064	Unity of land spatial planning	0.0030	0.4699
		Unity of planning management platform	0.0034	0.5301
Ecological environment protection system	0.0402	Unity of ecological protection laws and regulations	0.0107	0.2656
		Unity of ecological environmental standards	0.0170	0.4228
		Unity of ecological environment monitoring	0.0055	0.1373
		Unity of ecological environment law enforcement	0.0070	0.1744
Land management system	0.0017	Unity of construction land use planning	0.0004	0.2491
		Unity of the inventory land activation mechanism	0.0003	0.1573
		Unity of management of acquisition, storage and transfer of construction land	0.0010	0.5936
Factor flow system	0.0973	Unity of enterprise registration standards	0.0307	0.3156
		Unity of enterprise service supervision system	0.0092	0.0941
		Unity of mutual recognition and sharing of talent qualifications	0.0158	0.1627
		Unity of cross regional transaction of element resources	0.0416	0.4276
Financial and tax management system	0.0302	Unity of tax collection and management	0.0096	0.3181
		Unity of Cross Regional Sharing of Finance and taxation	0.0206	0.6819
Public service policy system	0.0357	Unity of public service standards and systems	0.0119	0.3333
		Unity of public service sharing	0.0238	0.6667
Public credit management system	0.0411	Unity of public credit evaluation system	0.0128	0.3125
		Unity of regional credit reward and punishment system	0.0283	0.6875
Jiangnan Water Town cultural brand	0.0544	Integrated development of humanity and nature	0.0323	0.5936
		Traditional culture development project	0.0131	0.2400
		Eco-cultural tourism development	0.0090	0.1663
Beautiful and livable rural environment	0.1606	Construction of beautiful countryside and boutique village	0.0435	0.2713
		Rural ecological environment governance	0.1170	0.7288
Interconnection infrastructure system	0.0080	Construction of transportation network system	0.0044	0.5531
		New infrastructure construction	0.0021	0.2621
		Intelligent construction and Application	0.0015	0.1848
High-level public service guarantee system	0.0629	Supply of public service resources	0.0157	0.2500
		Facilitation level of social security services	0.0472	0.7500

### 4.2. Analysis of the results

From the level of secondary indicators in Table 3, the top five secondary indicators in the weight of the high-quality development assessment system of the integrated demonstration area are ecological environment governance (0.2252), beautiful and livable rural environment (0.1606), innovation development level (0.1071), factor flow system (0.0973) and green economy business form (0.0680). Ecological environment governance is to give priority to ecological protection, and give play to the potential social and economic value of ecological green through the development and utilization of eco-tourism resources; Building a beautiful and livable rural environment is to promote the integration and infiltration of urban and rural development by creating a rural revitalization chain and creating a high-quality residential environment; The integrated construction of the demonstration zone promotes the agglomeration of various innovative resource elements in the region through system and mechanism innovation and environment creation, plays a role in the incubation, transformation and application of innovative achievements, and produces “aggregate effect”. The unified factor flow system can save transaction costs, but also facilitate industrial agglomeration, thus creating a good business environment for integrated high-quality development; Green economy formats can closely combine the characteristics and advantages of ecological green and traditional industries, transform industrial growth poles, form new growth drivers, and provide new ideas for the sustainable development of integrated demonstration areas.

Among the above five secondary indicators, the tertiary indicators with the highest proportion of internal local weights are the excellent water quality rate (0.7040), rural ecological environment governance (0.7288), innovative enterprise introduction (0.4673), cross regional transaction unity of factor resources (0.4276), and development level of new agricultural formats (0.4633), it shows that the integrated demonstration area should grasp the mature model of basin ecological coordinated governance, and achieve high-quality development of the integrated demonstration area through modern rural construction, the introduction of innovative enterprises, and the integration of trans regional factor transactions.

### 4.3. Formation of evaluation indices

According to the evaluation index system, two evaluation indices are constructed to evaluate the overall situation and individual differences of high-quality development in the integrated demonstration area.

(1) Comprehensive evaluation index (CEI) for high-quality development of integrated demonstration area. The comprehensive evaluation index is a technical treatment of quantifying the evaluation results. It is a weighted synthesis of multiple indicators to form a general index. The purpose of evaluation is achieved through the comparison of indexes. The four dimensions in the high-quality development evaluation system of the integrated demonstration zone can be combined into an index separately, and then a total index can be weighted based on the weights determined in this study to form a comprehensive evaluation index. The index can be used for horizontal comparison to evaluate the comprehensive development level of each region; Vertical comparison can also be conducted to evaluate the development and changes in different years.
(2) Differential diagnosis index (DDI) of high-quality development in integrated demonstration areas. The construction of differential diagnosis index is to analyze the degree of differentiation of the development level of “two districts and one county” in the integrated demonstration area and evaluate the degree of coordination of the integrated development of the demonstration area. In this study, the differential diagnosis index is constructed by the ratio of the regional single index range (maximum minus minimum) to the index average. The index can not only study the differences among indicators at all levels, so as to analyze the specific development gap of each region and point out the direction for the subsequent development; Diversified extension research can also be conducted to analyze development differences in other directions, such as comprehensive development level and green development level.

## 5. Conclusion and application

This study combed the connotation of high-quality development, green development and regional integration, respectively, and found the correlation between the three. Based on the analysis of the main content and indicator composition of the high-quality development evaluation system in the integrated demonstration area, the index system and model of the evaluation system were constructed, and the index weight of the model was further determined with the help of super decision-making software. This model provides a specific method for the subsequent actual evaluation of the development process and achievements. In the follow-up study, the model can be used to calculate the ecological, economic, social and other construction achievements of the integrated demonstration area by collecting relevant data of the integrated demonstration area. This research has high application value in two aspects: (1) taking the ecological green integration demonstration area in the Yangtze River Delta as the research object, applying the network analytic hierarchy process, establishing a high-quality development evaluation index system that comprehensively covers the three spaces of ecology, life and production in the integrated demonstration area, and providing methods and ways to measure the phased achievements of high-quality development and construction; (2) the indicator system can provide reference for the high-quality development evaluation research of other urban agglomerations or economic belts with coordinated development.

Based on the analytic network process, this study built an evaluation model for the ecological green and high-quality development of the Yangtze River Delta Demonstration area, which has achieved certain research results. However, due to the availability of data, there is still room for further improvement in this paper. In the future research, the model can be used to evaluate the high-quality development level of the demonstration area through the relevant data of the demonstration area.

## Data availability statement

The raw data supporting the conclusions of this article will be made available by the authors, without undue reservation.

## Author contributions

Conceptualization and writing—review and editing: LC and XY. Methodology and writing–original draft preparation: WW. Software and investigation: LC and WW. Validation and formal analysis: XY and WW. Resources, visualization, and supervision: LC. Funding acquisition: XY. All authors contributed to the article and approved the submitted version.
